# Penile Corporeal Reconstruction during Difficult Placement of a Penile Prosthesis

**DOI:** 10.1155/2008/370947

**Published:** 2008-11-04

**Authors:** Viet Q. Tran, Timothy F. Lesser, Dennis H. Kim, Sherif R. Aboseif

**Affiliations:** Section of NeuroUrology and Reconstructive Surgery, Department of Urology, Kaiser Permanente Medical Center, 4900 Sunset Boulevard, Los Angeles, CA 90027, USA

## Abstract

For some patients with impotence and concomitant severe tunical/corporeal tissue fibrosis, insertion of a penile prosthesis is the only option to restore erectile function. Closing the tunica over an inflatable penile prosthesis in these patients can be challenging. We review our previous study which included 15 patients with severe corporeal or tunical fibrosis who underwent corporeal reconstruction with autologous rectus fascia to allow placement of an inflatable penile prosthesis. At a mean follow-up of 18 months (range 12 to 64), all patients had a prosthesis that was functioning properly without evidence of separation, herniation, or erosion of the graft. Sexual activity resumed at a mean time of 9 weeks (range 8 to 10). There were no adverse events related to the graft or its harvest. Use of rectus fascia graft for coverage of a tunical defect during a difficult penile prosthesis placement is surgically feasible, safe, and efficacious.

## 1. INTRODUCTION

For some patients with both impotence and concomitant penile fibrosis, placement of a penile prosthesis is the only viable therapy to allow restoration of erectile function. Placement of a penile prosthesis in the setting of severe corporeal or tunica albuginea fibrosis can be very difficult and challenging. This may hinder the surgeon from satisfactorily dilating the corporeal bodies to accommodate the prosthesis and/or closing the tunica albuginea over the cylinders. This in turn may negatively affect the function of the prosthesis, limit the size of the prosthesis, and ultimately negatively impact the patient's overall satisfaction [1].

Several etiologies of corporeal fibrosis have been identified including multiple penile surgeries, prior removal of an implant for infection, erosion or malfunction, priapism, chronic intracavernous injections, penile trauma, or Peyronie's disease [2–5]. In phalluses with extreme fibrosis where satisfactory dilatation of the corpora and/or closure of the tunica albuginea is not feasible, several techniques have been described to allow for placement of a penile implant. One option is to use readily available downsized implants [6]. The Otis urethrotome has been used to perform an extended corporotomy with extensive tunical excision to facilitate placement of an implant [7]. When these various techniques are unsuccessful, penile reconstruction with or without graft material is necessary [8].

Grafting of the penile corpora is a valuable tool that can help surgeons in penile reconstructive surgery. In 1950, Lowsley and Boyce described the first usage of a fat graft to surgically repair Peyronie's disease [9]. Since then, various grafts have been explored in search of an ideal graft that best mimics the properties of the tunica albuginea. These characteristics should include good compliance and pliability, minimal inflammation, high tensile strength to prevent bulging or aneurysmal dilatation, low antigenicity risk, low infection transmission risk, availability in various sizes, packaging, and cost [10]. Modern graft materials described in the literature include fat, vein, rectus fascia, tunica vaginalis, temporalis fascia, dermis, cadaveric dura, cadaveric pericardium, porcine small intestine submucosa (SIS), and synthetic grafts such as Dacron and Gore-Tex [11–20]. Choosing an appropriate graft material and technique is a crucial aspect for successful tunical/corporeal reconstruction and ideal functional outcome of prosthetic surgery.

The use of rectus fascia is well documented in the literature for a variety of uses in reconstructive surgery. As we previously reported, we evaluated the functional outcomes and patient satisfaction in patients who underwent rectus fascia grafting for reconstruction of the corporeal bodies in the setting of severe tunical/corporeal fibrosis in order to facilitate the placement of a penile prosthesis [21].

## 2. MATERIAL AND METHODS

As previously reported, 15 patients who underwent placement of an inflatable penile implant and corporeal/tunica reconstruction using autologous rectus sheath were included into the study [21]. The patients were divided into two groups. *Group I* included seven patients who had tunica fibrosis secondary to Peyronie's disease and associated severe erectile dysfunction. These patients had penile curvature and erectile dysfunction for more than 12 months (mean 14 months) and had exhausted all medical forms of treatment. None in this group had prior surgery for correction of penile curvature or impotence. *Group II* was composed of eight patients who had severe corporeal fibrosis related to a history of penile prosthesis removal secondary to malfunction (*n* = 1), infection (*n* = 3), or erosion (*n* = 4). Patients had their devices placed at least 24 months prior to reinsertion (mean 36 months). Six of these eight patients had more than one implant surgery in the past.

All patients in *groups I* and *II* underwent placement of a three-piece inflatable penile prosthesis. Seven were recipients of a Mentor Alpha I inflatable penile implant and the other eight were recipients of an AMS 700 inflatable implant. Patients in both groups had a significant tunical albuginea defect after the placement of the prosthesis, requiring corporeal reconstruction with graft material to provide adequate corporeal coverage and closure. The defect size was determined at the time of surgery and a corresponding sized graft was harvested from rectus fascia. Postoperative evaluations and exams focused on the function of the prosthesis, with attention to any findings suggesting herniation or erosion. Also, patients' abdomens were closely examined for any evidence of hernia or wound infection related to the graft harvest site. Follow-up clinical exams and interviews were conducted at 1, 6, and 12 months, and yearly visits thereafter.

### 2.1. Surgical technique

The surgical technique is detailed here as we have previously described [21]. Preoperative intravenous antibiotics, including 1 gram of Vancomycin and 160 mg of Gentamicin (adjusted for patient weight and renal function), were administered to all patients. Patients were positioned supine on the operating room table. A 16 French Foley catheter was inserted prior to making the skin incision.

**Group I**

**Figure 1:**
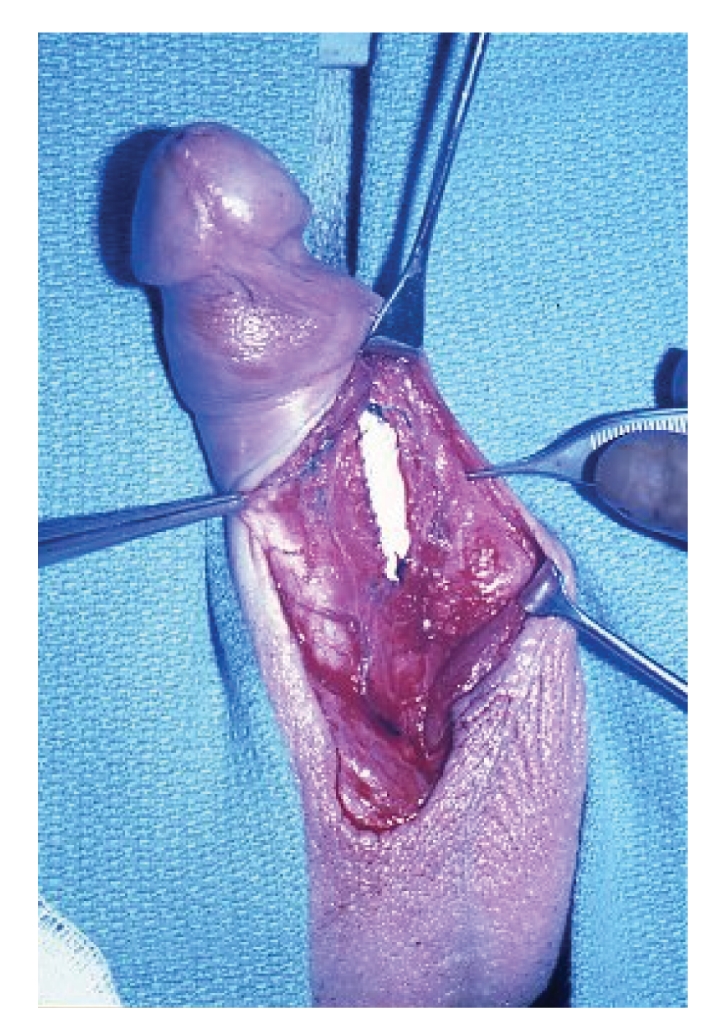
Augmentation of the tunica albuginea is shown here with a rectus fascia graft sewn in place allowing coverage of the penile prosthesis.

After making a circumcision incision, the penile skin was degloved exposing both corpora cavernosa and neurovascular bundles. An artificial erection was induced using injectable saline with compression at the base of the penis. Both neurovascular bundles were dissected and reflected laterally and the tunical plaque was exposed. The point of maximum curvature was marked and an H-incision was made at this site: two lateral, longitudinal incisions for placement of the prosthetic cylinders, and a transverse incision on the plaque to release the curvature as previously described by Aboseif et al. [22]. After corporeal dilation and proper sizing of the implant, the rectus fascial graft was harvested through a transverse suprapubic incision. The grafts were excised as rectangular strip to correspond with the tunica albuginea defect size (mean 2 cm × 8 cm). The reservoir was then placed in the retroperitoneal space through the same incision. The graft was then fashioned to cover the defect, and secured using 4-0 Maxon suture in a running fashion (Figure 1). Caution was taken to prevent injury to the underlying inflatable implant during this step.

**Group II**

A midline penoscrotal incision was made to expose both corpora cavernosa. A longitudinal incision was made on the ventrolateral surface of each corpus, extending from the glans penis to the most proximal position possible. Caution was taken to avoid cutting through the full thickness of the corporeal bodies, as there is usually dense scar tissue obliterating the normal planes and possibly the intracorporeal tissue. Once in good position, the intracorporeal space was dilated both proximally and distally using Metzenbaum scissors. After proper sizing of the implant, the tunical defect was measured, and the rectus fascia was harvested and secured with 4-0 Maxon as described earlier.

This technique is similar in concept to an on-lay urethroplasty for urethral stricture disease. By incising the tunica and placing a graft, the size of the corporeal bodies conceivably is enlarged which allows its closure without any tension or ischemia to the tissues.

Once the tunical defect was closed with the rectus fascia graft, inflation of the prosthesis was performed to ensure correction of the defect and proper function of the new prosthesis. The penile and the suprapubic wounds were closed in a normal fashion. Penile dressing and scrotal fluff were then applied.

Patients were admitted overnight for postoperative observation and continued on intravenous antibiotics. Average hospital stay was one day. The foley catheter was removed prior to the discharge. Patients were instructed to keep the device deflated for 6 weeks. Subsequent follow-up appointments were scheduled for 1-, 6-, and 12-month intervals, followed by yearly visits. Clinical data concerning the prosthesis function, integrity of the graft, wound healing, patient satisfaction and complications were evaluated during each follow-up visit.

## 3. RESULTS

Fourteen out of fifteen patients were available for evaluation, while the remaining patient was lost to follow-up. Placement of a functioning inflatable penile prosthesis with reconstruction of the tunical deficiency with autologous rectus fascia was successful in all 14 patients at a mean follow-up of 18 months (range 3 to 36). All of the implanted prostheses were functioning appropriately, with all 14 patients reporting satisfactory sexual intercourse. Six patients reported suprapubic discomfort with moderate-level activity in the first 3–6 months, which resolved in 5 of the 6 patients. Four patients had penile hypoesthesia of the glans, which eventually resolved in 2 patients but persisted in the other 2. One patient complained of shortening of his penis, but he was still able to have satisfactory intercourse. There was no evidence of rectus fascia graft compromise since no kinking or herniation of the prostheses was found. Furthermore, there were no adverse events such as infection, prosthesis malfunction, incisional hernia, or fluid collections at the site of graft harvest that resulted from the harvesting of the rectus fascia.

## 4. DISCUSSION

In a subset of patients with impotence and concomitant severe tunical and cavernous tissue fibrosis, reestablishment of erectile function is dependent on implantation of a penile prosthesis. In this setting, a sizable tunical defect may be encountered during penile prosthesis implantation and corporeal reconstruction may become necessary. Several surgical techniques have been described to handle such situations. Corporeal reconstruction with graft material is an acceptable option. Various materials for such reconstructive repairs have been used and described in the literature.

The use of nonautologous grafts for corporeal reconstruction has been widely reported in the literature. Commercially available human cadaveric fascia or porcine tissues are viable options for graft material. These free tissue grafts have been used extensively in various urologic procedures for other applications such as placement of a pubovaginal sling or during the surgical correction of Peyronie's disease [23, 24]. The use of the various porcine tissue grafts (including dermis, pericardium, and small intestinal submucosal) has been published, showing good results [18, 25]. These graft materials provide off-the-shelf availability, decreased operative time, and no donor site morbidity, however they are quite expensive.

Synthetic grafts (i.e., Gortex, dacron, prolene) are readily available, come prepackaged in various sizes, and do not require a second incision to harvest from a donor site. The drawbacks to using a synthetic graft are that they are costly, may predispose the patient to infection, and behave physiologically different than the tunica albuginea [6, 26]. The tensile strength of the synthetic graft is much greater than the native tunica albuginea resulting in limited expandability. These characteristics limit the full expansion potential of the cylinders [27].

Harvesting autologous grafts is performed for various urologic and nonurologic reconstructive surgeries. These grafts include rectus fascia, fascia lata, dermis, saphenous vein, temporalis fascia, and tunica albuginea. They have the advantage of being noninfectious and nonimmunogenic, possess good tensile strength, and are readily abundant to close any size tunical defect. The potential disadvantages of using autologous grafts may include increased operative time secondary to harvest time, bleeding, and morbidity related to the harvest site [10]. Venous grafts have been shown to be superior to other autologous tissues in the repair of Peyronie's disease owing to its physiologic properties that better mimic the vascular intercorporeal space [28, 29]. It is more elastic and is less likely to contract than other autologous tissues, and the venous endothelium offers the theoretical advantage of nitric oxide secretion to maintain normal erectile physiology [28]. When tissue graft material is used to reconstruct the corpora in facilitating implantation of a prosthesis, reconstructing the corpora with tissue material that is nonsynthetic and has high tensile strength is crucial in providing adequate tissue support for the inflatable penile prosthesis. In our reported study, we elected to use autologous rectus fascia because it fulfills these requirements and it also allowed harvesting of the graft to occur through the same suprapubic incision that is made for the placement of the inflatable prosthesis reservoir without having to make a separate incision [21].

Using an autologous rectus fascial graft for corporeal reconstruction helps facilitate the placement of a penile prosthesis when encountering severe tunical/corporeal fibrosis. This is similar in concept to on-lay urethroplasty for stricture disease; the graft allows the overall corporeal body circumference to increase which in turn allows the implant to inflate better. Furthermore, it allows closure of the tunica without tension, thus avoiding any possible ischemia of the tissues with its risk of infection and complications. All patients in the study incurred no complications related to the use or harvesting of the graft. Long-term follow-up demonstrated that all the patients in the study had excellent prosthetic function and were satisfied with their overall outcomes.

## 5. CONCLUSION

We concluded in our study that the use of rectus fascia grafts for the augmentation of the tunical deficiencies and corporeal reconstruction during difficult penile prosthesis implantation yielded excellent clinical results. Long-term outcomes demonstrated high overall patient satisfaction. Ease of harvesting, reduced cost, elimination of the synthetic and xenographic materials make this graft an excellent anatomic and functional tunical substitute. Rectus fascia graft is a valuable addition to the reconstructive urological repertoire and should be considered when tunical defect precludes adequate tunical closure during penile prosthesis implantation.
